# Experimental Demonstration of the High Alignment-Tolerant Behavior of a Mid-Infrared Waveguide Platform for Evanescent Field Sensing

**DOI:** 10.1021/acsaom.4c00280

**Published:** 2024-08-20

**Authors:** Felix Frank, Mattias Verstuyft, Nuria Teigell Beneitez, Jeroen Missinne, Gunther Roelkens, Dries van Thourhout, Bernhard Lendl

**Affiliations:** † Institute of Chemical Technologies and Analytics, TU Wien, Getreidemarkt 9, 1060 Wien, Austria; ‡ Photonics Research Group, Ghent University-imec, Technologiepark-Zwijnaarde 126, 9052 Gent, Belgium; § Center for Microsystems Technology, Ghent University-imec, Technologiepark-Zwijnaarde 126, 9052 Gent, Belgium

**Keywords:** mid-infrared, waveguide, photonic integrated circuit, silicon photonics, alignment tolerances

## Abstract

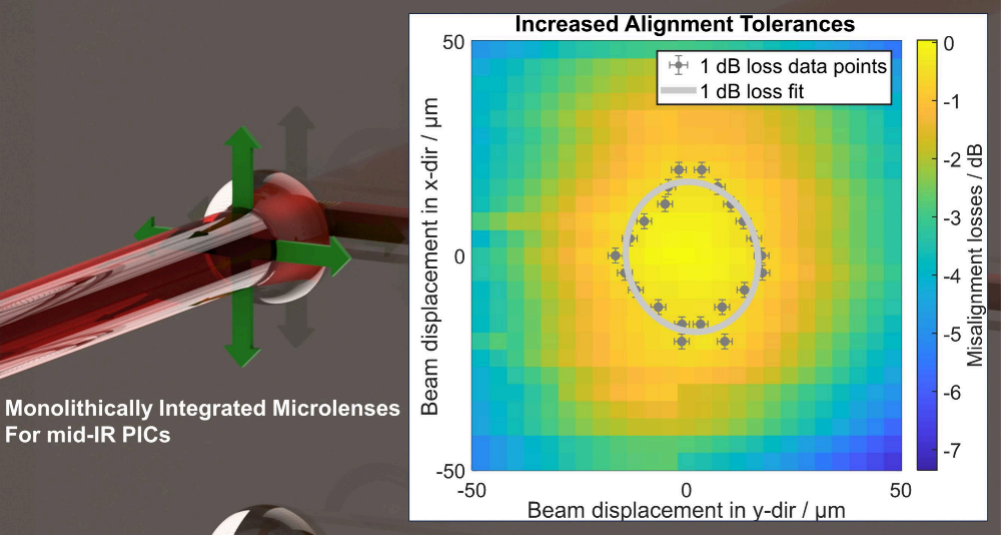

Alignment tolerant coupling interfaces are an important feat for mid-IR waveguides when moving closer to real-world sensing applications, as they allow for an easy and fast replacement of waveguides. In this work, we demonstrate the alignment tolerant behavior of a germanium-on-silicon trenched waveguide platform with monolithically integrated microlenses using backside coupling of an expanded beam for evanescent field sensing between 6.5 and 7.5 μm. The chip with a propagation loss of approximately 5 dB/cm was mounted and aligned, using active alignment, in a sample holder that could be moved in all three dimensions to induce misalignments with a precision of the manual actuator of 1.3 μm. Using this setup, the in-plane 1 dB alignment tolerances were measured to be ±16 μm, while the 1 dB alignment tolerances in the longitudinal direction were found to be larger than ±150 μm. Without the addition of the microlenses, we expect an in-plane 1 dB alignment tolerance of ±3 μm based on simulations. Additionally, it could be demonstrated that the integration of the microlenses significantly improves the stability of the broadband grating couplers in regard to misalignment-induced intensity changes in the obtained transmission spectra.

## Introduction

Silicon photonics (or group IV photonics) has evolved into a mature and widely adopted technology in the field of integrated photonics. This has led to the establishment of highly reliable large-scale sites for the production of photonic circuits in a compact and cost-efficient way for established materials such as silicon on insulator (SOI). Recently, the use of germanium (Ge) and silicon (Si) hybrid materials has increased, especially in the field of mid-infrared (IR) spectroscopy, where the use of SOI is limited by the low-loss transmission range of 1.2–6 μm.^1^ Germanium, on the other hand, is a close to perfect material for the use in photonic systems in the mid-IR range, as it is transparent in nearly the entirety of the mid-IR region and has a high refractive index of 3.97 at 5 μm (compared to 3.47 for Si),^2,3^ but does not offer the same mature fabrication sites compared to SOI. Subsequently, the first mentions of the high theoretical efficiency of germanium-on-silicon (GOS) materials were found in the mid-2000s,^1^ followed by the demonstration of early devices some years later, proving their low loss properties in the mid-IR.^4−6^ Most early works focused on growing Ge ribs onto the surface of either SOI wafers or etching rib waveguides into Ge-on-insulator wafers.^7,8^ Further investigations based on these rib structures have then isolated the mode confinement in the interlayer between Ge and Si as one of the major reasons for the propagation loss of these waveguides.^9^ This is due to the mismatch of the lattice constant of 4% between Ge (5.66 Å) and Silicon (5.43 Å) which results in imperfect crystal growth in the Ge through dislocation and threading defects.^10^ Another source of loss for Ge grown on Si is due to unintended background doping of the Ge, resulting in high propagation losses due to free-carrier absorption.^11^

To minimize these losses, new strategies were proposed, such as going to GOS wafers and etching the waveguide structures into the top germanium layer. By doing this, a layer of Ge between the waveguide and the Ge–Si interface, limiting the propagation losses caused by the mode confinement in the Ge–Si interlayer.^12^ By properly tailoring the deposition parameters during the Germanium growth, free carriers can also be suppressed.^13^

Using this technique, propagation losses of ∼1 dB/cm could be demonstrated in the mid-IR (for wavelengths of 10 μm^13^ and 5–8 μm,^14^ respectively), moving closer to practical sensing application of these waveguides.

Looking beyond the inherent propagation losses, which can be minimized by design and fabrication of the waveguide structures, the second considerable source of loss when operating waveguides are coupling losses. This is especially true for silicon photonics, as its compatibility with current optical fiber components is not optimal, making high-efficiency coupling still challenging.^15^ Conceptually, two main strategies are chosen to couple light into waveguide structures, edge-coupling and vertical coupling. Edge coupling shows very high coupling efficiency and a relatively high bandwidth, while traditional grating couplers struggle to achieve both feats at the same time.^16,17^ On the other hand, edge couplers need to be placed at the edge of the chip, whereas grating couplers can be placed anywhere on the chip, giving more flexibility in design, and making fabrication significantly easier. In addition, they generally exhibit wider alignment tolerances than edge couplers, which is crucial for bringing integrated waveguide optics closer to industrial applications.^17,18^ By integrating monolithic back-side microlenses, the alignment tolerances can be improved even further, which has been first demonstrated for the NIR with 1 dB alignment tolerances of ±10 μm.^19,20^ By using an expanded beam for coupling, which is focused onto the gratings by means of silicon lenses, this concept has also been applied to the mid-IR.^20,21^ Building on these findings, we demonstrated an integrated circuit platform using trenched GOS waveguides employing back-side coupling with monolithically integrated microlenses and broadband grating couplers with a 3 dB bandwidth of ∼500 nm at the cost of a decreased coupling efficiency of 30%, and slightly increased simulated back reflection of ∼20% in our previous work. However, no impairment of the laser operation was observed due to back-reflections. We further combined these waveguides with functional mesoporous layers for analyte enrichment in the evanescent field to increase sensitivity, reaching limits of detection of 7 ppm for toluene in water.^22^

In this work, we focused on the experimental demonstration of the alignment tolerances of our previously reported waveguide platform to showcase its potential for a range of practical applications. We mounted the chip on an xyz-stage with manual adjusters, with a scanning range of 100 μm × 100 μm for in-plane alignments and ±150 μm for out-of-plane alignments. Using this setup, we looked at relevant properties for PICs when going toward practical applications, demonstrating the advantages of monolithically integrated microlenses in the mid-IR. First, we compared the experimental alignment tolerances of the entire PIC waveguide platform to the simulated alignment tolerances of the grating. We then investigated the stability of the transmission spectrum for different alignments, showing that spectral shifts caused by grating coupler misalignments can be significantly reduced. The results of this experimental demonstration can serve as a catalyst for further research in the emerging field of mid-IR photonics, bringing mid-IR waveguides closer to application.

## Results and Discussion

### Experimental Determination of the Alignment Tolerances

**Figure 1:**
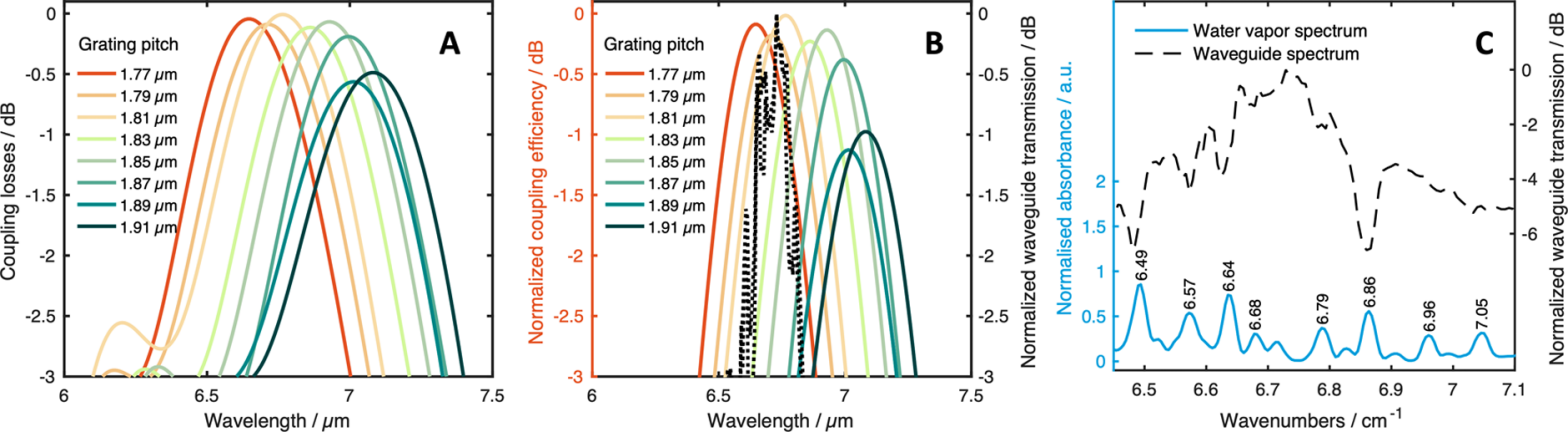
(A) Simulations of the normalized coupling losses of the gratings for different grating pitches. (B) Simulations for the normalized coupling efficiency (in- and out-coupling) and the waveguide transmission spectrum (black dotted line). (C) Water vapor spectrum and waveguide transmission spectrum between 6.45 and 7.15 μm showing the influence of water vapor in the recorded transmission spectrum.

To determine the alignment tolerances of the waveguide platform, we swept the in-plane directions systematically. Initially, the setup was actively aligned using the detector feedback to achieve optimal alignment, which is further referred to as ’reference point’. This optimal alignment was achieved for a grating pitch of 1.79 μm. Simulations of the normalized coupling losses, the normalized coupling efficiency and the transmission spectrum of the waveguide suggesting alignment at a grating pitch of 1.79 μm are shown in Figure 1. The influence of water vapor on the transmission spectrum is shown in Figure 1C, as the dips in transmission at 6.64, 6.68, 6.71, 6.79, and 6.85 μm can be assigned to the water vapor absorption bands. As we were using an expanded beam concept, the angular tolerance of the chip needs to be considered. For this, a chip holder as described in our previous report^22^ was used to keep the chip in place and limit angular misalignments. Similarly, potential misalignments between the microlens and the grating couplers must be considered, as they can impose angular misalignments. This, however, can be limited by precise fabrication, and can be neglected from a practical standpoint.

**Figure 2:**
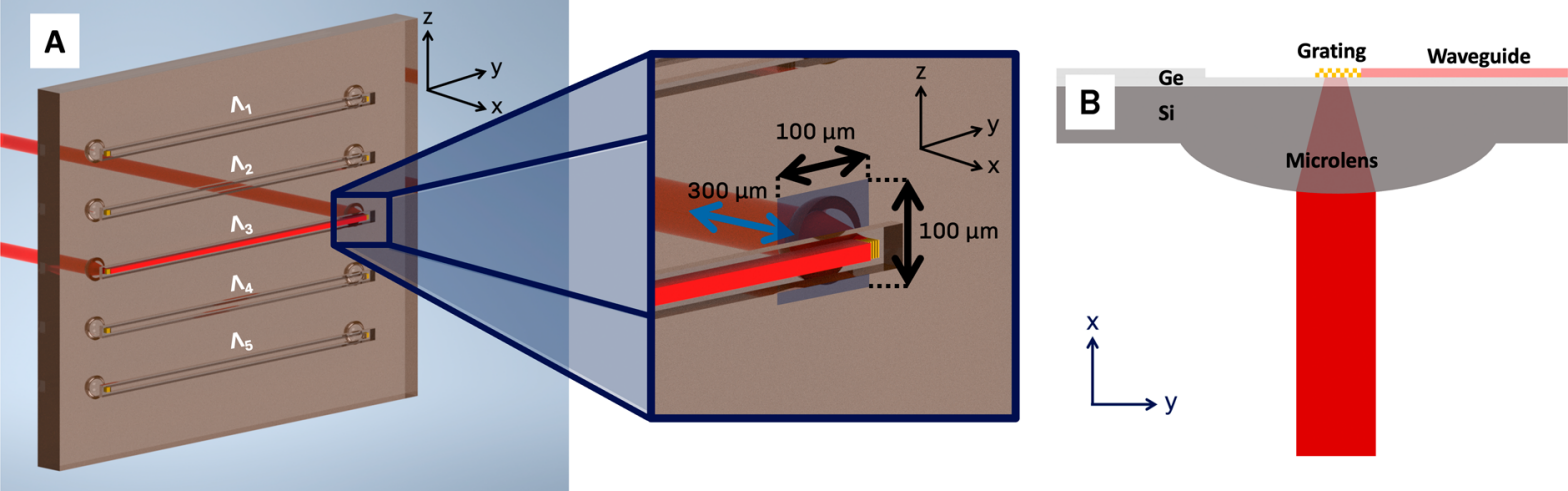
(A) Schematic depiction of the waveguide chip (Λ_n_ denoting different grating pitches for each waveguide) and the scanned area (blue area marked on the chip, in-plane movement marked with black arrows, out-of-plane movement marked with blue arrows). (B) Schematic depiction of the cross-section of the waveguide, demonstrating the vertical coupling concept of the integrated microlenses and grating couplers.

Subsequently, we performed a scan of a 100 μm × 100 μm area centered at the reference point, with 4 μm increments in both the y- and *z*-directions, capturing a spectrum at each point. This involved taking a reference spectrum, followed by sweeping in *y*-direction while maintaining a fixed position in the *z*-direction. To assess the out-of-plane alignment tolerances, we acquired spectra in-axis relative to the reference point, utilizing the entire 300 μm fine travel range of the manual adjuster (schematic depiction of the waveguide chip and the scanned area shown in Figure 2). Each spectrum was obtained by averaging 1000 individual spectra, with a total integration time of 1 s.

**Figure 3:**
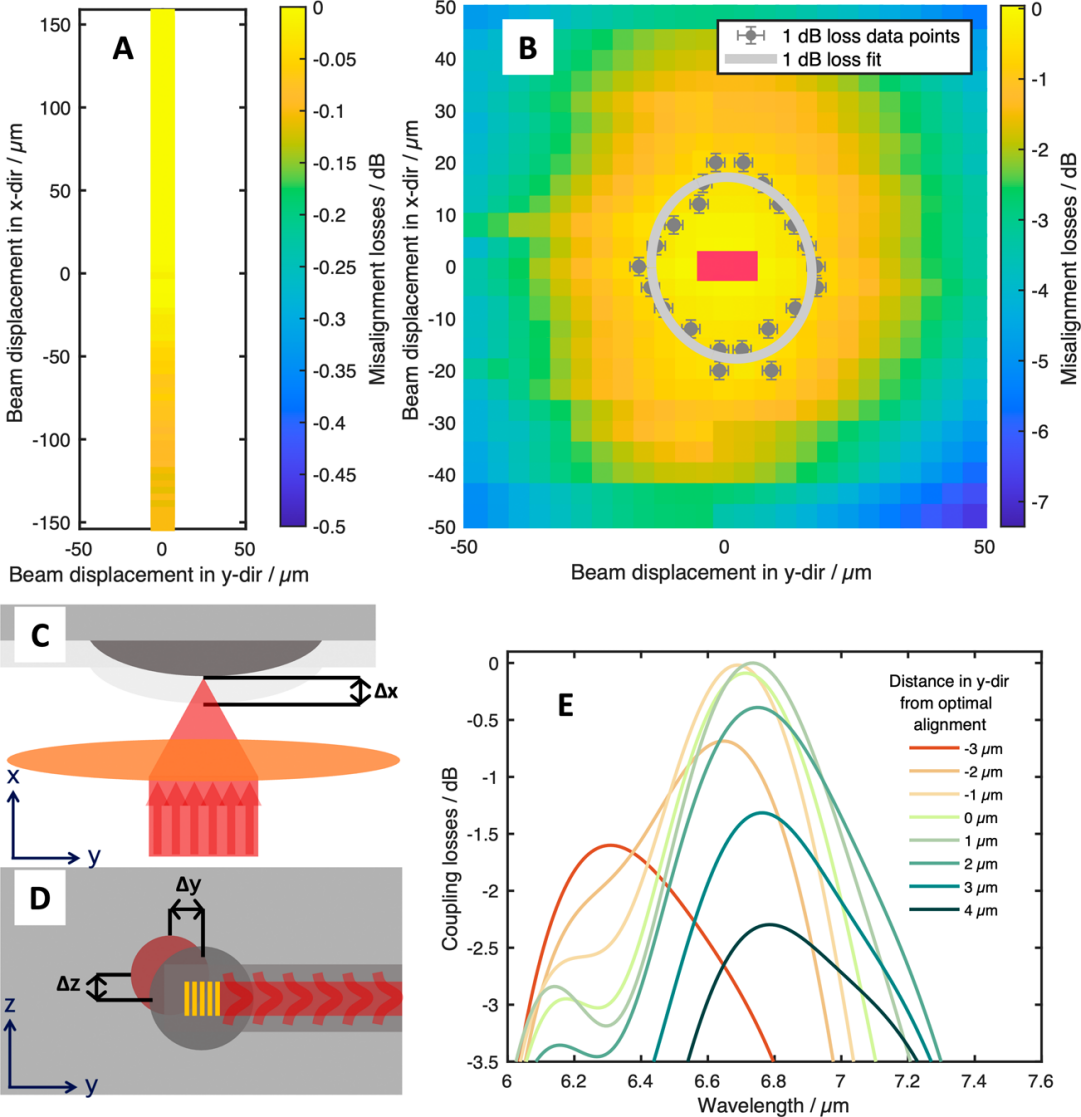
(A, B) Experimental alignment tolerances of the waveguide platform in regard to misalignments in *x*- and *y*-directions (A) and *y*- and *z*-directions (B). Each measurement series for the respective misalignment in *z*-direction was fitted with a Gaussian curve, with the 1 dB loss marked by the gray data points and the 1 dB loss fit for total beam marked by the gray ellipse. Area covered by the simulations in E marked with pink box. (C) Schematic depiction of misalignments in *x*-direction. (D) Schematic depiction of misalignments in *y*- and *z*-directions of the laser (red circle) relative to the microlenses (gray circle) and the grating couplers (yellow structure). (E) Simulated transmission spectra for different misalignment distances in *y*-direction relative to the center of the grating coupler for a grating pitch of 1.79 μm.

For the out-of-plane scans (*x*-axis), in which the focus of the beam was changed by moving the chip in longitudinal direction relative to the fixed focusing lens, no significant misalignment losses could be found, with the whole fine travel range (±150 μm) of the manual adjuster leading to maximum losses <0.2 dB, as seen in Figure 3A. This was to be expected as the calculated beamwidth at 150 μm longitudinal misalignment is only 43 μm (compared to a beam waist of 7.5 μm and a radius of curvature of the lens of 530 μm). To determine the 1 dB alignment tolerances, the single channel spectra of the reference point and sample points were fitted with Gaussian profiles to mitigate the influence of slightly changing water vapor concentrations. The losses were determined as the ratio of the maximum of the fitted sample spectra compared to the maximum of the reference point. In Figure 3B, the mapping of all in-plane spectra is shown. For each sweep in the *x*- and *y*-dimensions, the data points were fitted with another Gaussian profile, and the 1 dB loss misalignment points were determined (shown as gray points, error bars show the influence of noise). The 1 dB alignment tolerance of the platform was determined as the mean distance of the 1 dB loss misalignment points to the reference point, which equated to 16.4 μm (compared to <3 μm for the simulated values for the grating couplers). This is approximately half of the simulated 1D-behavior of ∼30 μm, but slightly better than the experimental 1D hollow-core fiber results presented in our previous report.^22^ Adding to this, it also has to be said that the simulations do not take into account the change of the incidence angle for lateral misalignments, which may add slightly to the worse performance when comparing the experimental results to the simulations, although the angular dependence of the coupling efficiency of the employed grating couplers is rather low. The better experimental alignment tolerances may be attributed to a better focusing of the IR beam using free space optics, as hollow-core fibers with an output beam diameter of 130 μm were used for in-coupling in our previous report.

### Experimental Determination of Misalignment Induced Changes of the Transmission Spectra

**Figure 4:**
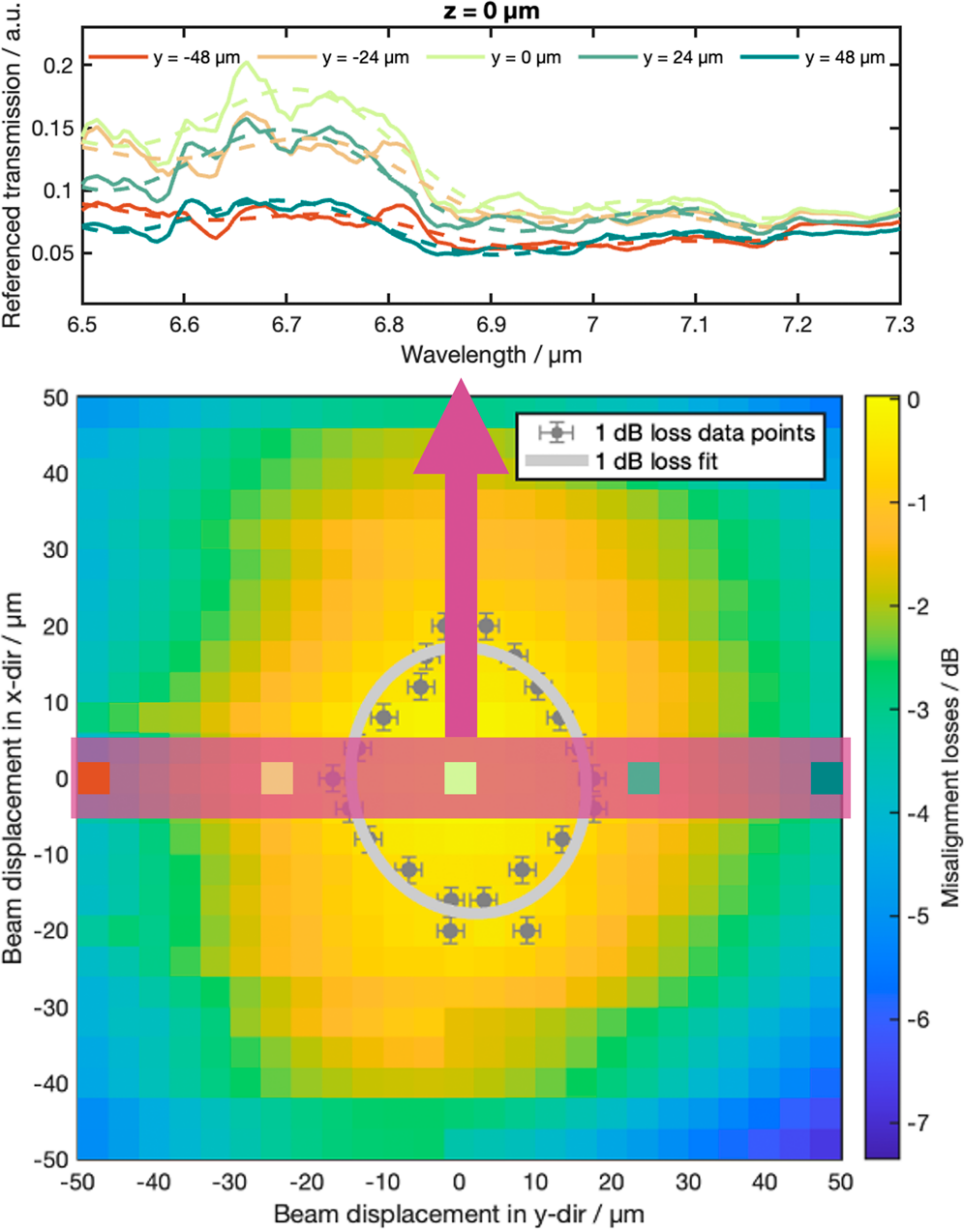
(A) Referenced transmission spectra (solid lines) and Fourier fits (dotted lines) at different misaligned points in *y*-direction. (B) Locations of the spectra are shown in the 1 dB loss map.

In Figure 4, transmission spectra at different alignment positions can be seen. In contrast to the single line spectra used for Figure 3, these spectra are calculated as transmission spectra referenced to the power spectrum of the spectrometer. Looking at the spectra, apart from the obvious decrease in coupling efficiency when moving away from the center, it can be noted that the transmission spectrum changes, especially when moving in the *y*-direction (perpendicular to the orientation of the grating).

**Figure 5:**
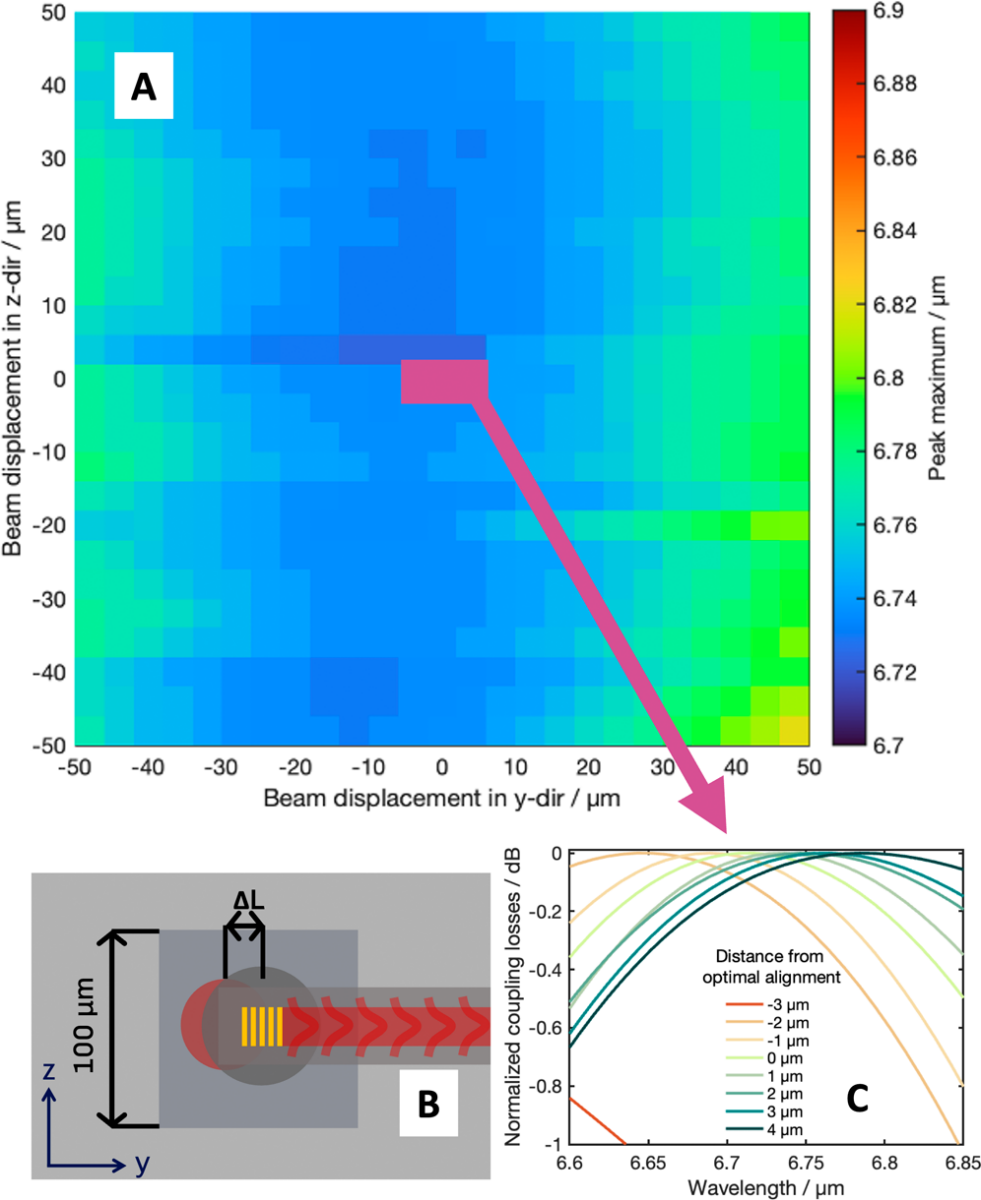
(A) Experimental peak maximum of the referenced spectra. The borders of the color bar refer to the two simulated peak maxima of the grating couplers for different alignments. The pink area shows the region of the simulation for the grating couplers. (B) Schematic depiction of misalignments of the laser (red circle) relative to the microlenses (gray circle) and the grating couplers (yellow structure). (C) Normalized simulated transmission spectra for different misalignment distances in *y*-direction relative to the center of the grating coupler for a grating pitch of 1.79 μm.

To investigate any spectral shifts induced by misalignments, two approaches were chosen: The first one was identifying the wavelength with the highest transmission, while the second approach studied parameter concerned the change of the transmission spectrum shape. For that, the calculated chip transmission spectra (Figure 4A, solid lines) were fitted with a two term Fourier function (Figure 4A, dotted lines) in the spectral range of the spectrometer between 6.5 and 7.2 μm. The use of a Fourier fit was due to the dip in transmission at 6.85 μm caused by strong water vapor bands (seen in Figure 1C) to still obtain a satisfying fit. The maxima of these function were then mapped and are shown in Figure 5. Looking at this map, the maximum of the peak does not seem to shift in the scanned 100 μm × 100 μm area. Comparing this to the simulations of the shift of the grating coupler (Figure 5C), it can be seen that integrating the microlenses leads to a much more stable peak maximum for the waveguide transmission spectra, as the simulations for the grating couplers suggest wavelength shifts at lateral misalignments of approximately 3 μm. Considering industrial applications, where not only the coupling losses, but also the wavelength range, needs to be stable in an environment, where slight misalignments are inevitable, this demonstrates the capabilities of the presented waveguide platform in that concern.

**Figure 6:**
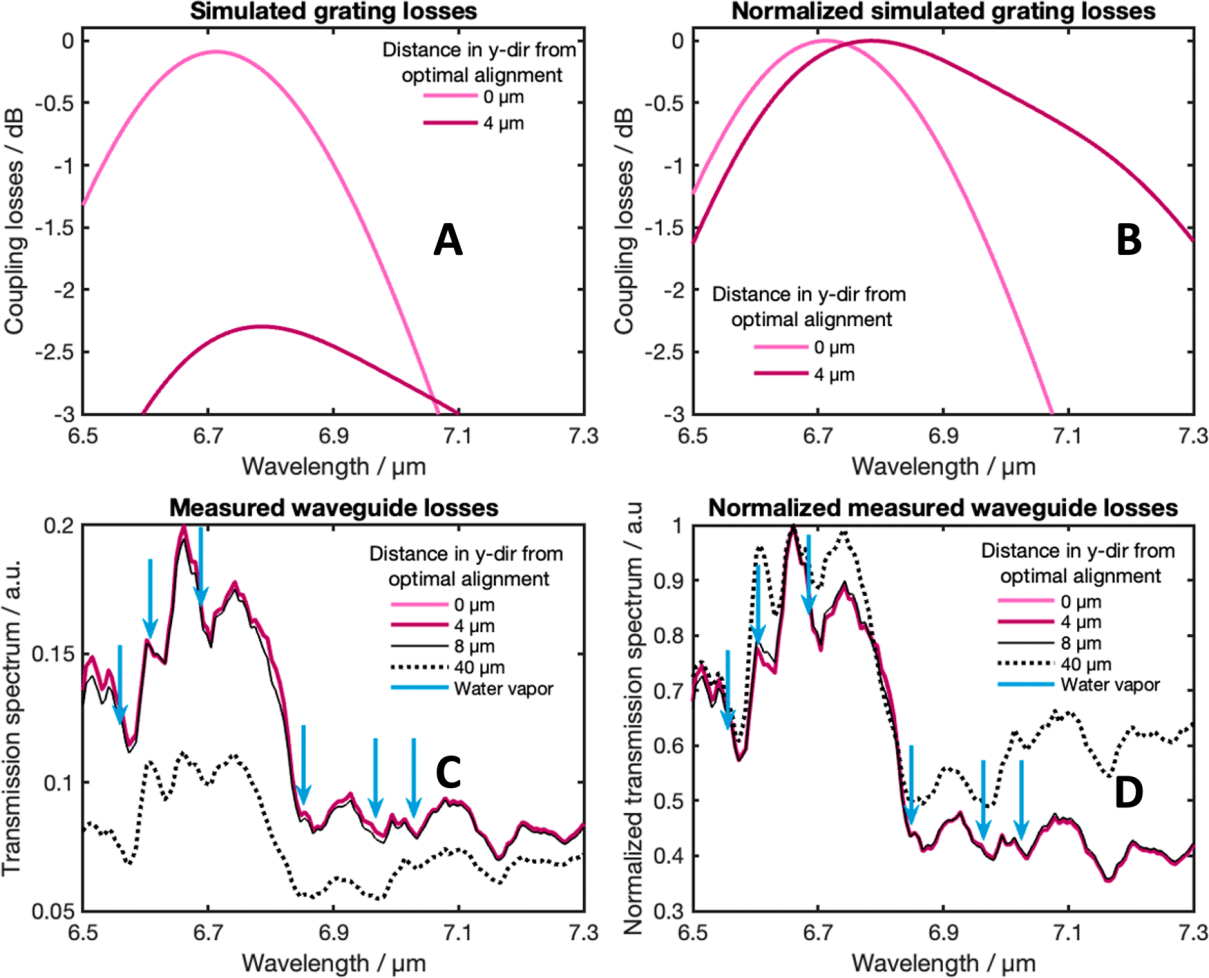
(A) Simulated grating losses for an optimal alignment and a misalignment of Δ*x* = 4 μm. (B) Normalized simulated grating losses for an optimal alignment and a misalignment of Δ*x* = 4 μm. (C) Measured waveguide transmission spectra for an optimal alignment and misalignments of Δ*x* = 4, 8, and 40 μm. Water vapor band positions are shown with red lines. (D) Normalized measured waveguide transmission spectra for an optimal alignment and misalignments of Δ*x* = 4, 8, and 40 μm. Water vapor band positions are shown with blue arrows.

**Figure 7:**
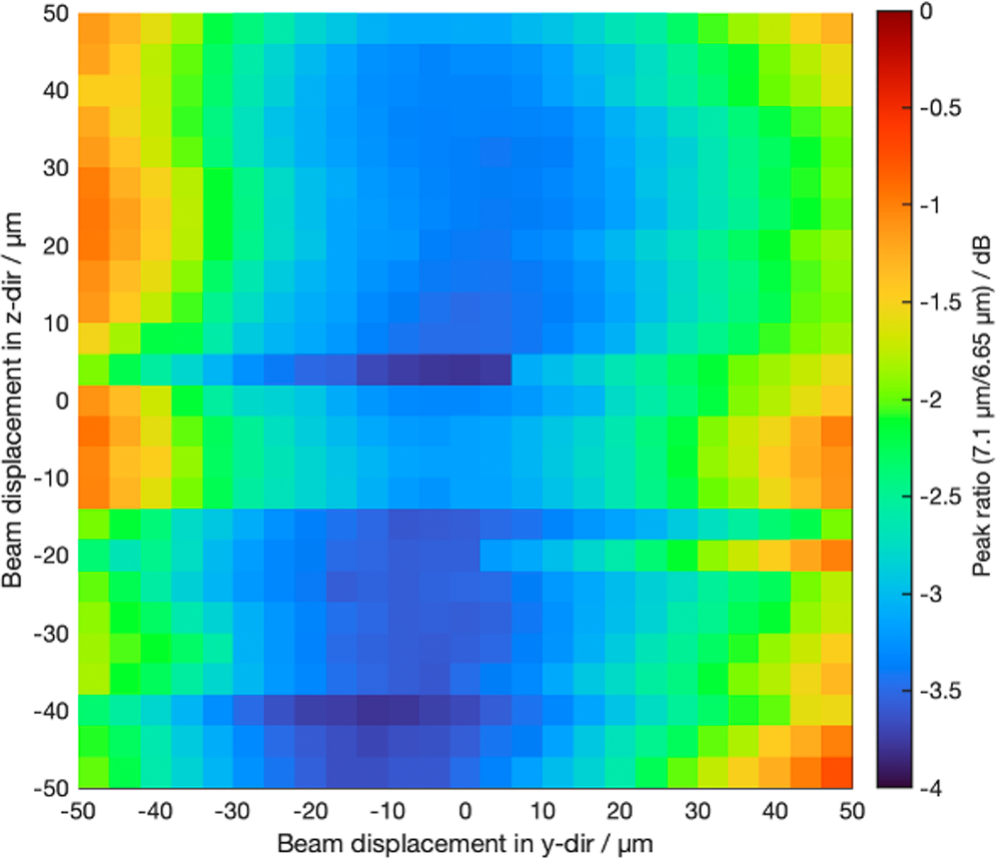
Experimental ratio of the peak maximum at 6.65 μm and the growing peak shoulder at 7.1 μm of the grating couplers.

As the peak maximum did not change significantly across the scanned area, we adopted a different approach to detect more subtle variations in the spectral features of the waveguide transmission spectra. In Figure 6, we compare the simulated grating losses for different misalignments with the corresponding measured spectra. Two important observations emerge from this comparison: First, the alignment-tolerant behavior of the waveguide is showcased in the transmission spectra, as a misalignment of 4 μm does not result in a significant change in the transmission. In contrast, the simulation predicts a similar decrease in transmission for a 4 μm misalignment as it was observed for a 40 μm misalignment in the measured data. Second, the normalized measured transmission spectra exhibit broadening toward higher wavelengths. Notably, strong water vapor bands exist in the region between 6.8 and 7 μm, making this broadening most evident between 7 and 7.3 μm. Consequently, we calculated the ratio of transmission spectra at 6.65 and 7.1 μm as a measure of the misalignment-induced changes in waveguide transmission. Figure 7 presents a map of this ratio. Upon closer examination, we observe a slight red shift in the transmission spectra near the edge of the grating couplers, suggesting peak shifts. However, this shift does not align with lateral misalignments of less than 4 μm, as predicted by the simulations. Hence, the high margin of error for in-plane misalignments, especially perpendicular to the propagating direction of the waveguide further showcases the increased alignment tolerance of the microlens system when comparing it to a simulation only including the grating couplers.

## Experimental Section

The fabrication of the waveguide chips can be divided into the fabrication of the waveguide structures with grating couplers and the subsequent fabrication of the microlenses on the backside and is described in detail in our previous report.^22^ The sensing chips consist of trenched rib waveguides with a width of 3.3 μm (at a trench width of 8 μm), a distance of 16 mm between the grating couplers, and an etching depth of 1 μm on a 2 μm GOS platform. The grating couplers support TE polarization and have 8 different grating pitches for each waveguide on a chip (between 1.77 and 1.91 μm) and are designed to feature a high bandwidth and low angular dependency. In order to focus the incident laser beam and to collimate the emitted output beam, microlenses were etched into the backside of the chip, aligned directly below the grating coupler. The radius of curvature of the microlenses is in line with our previous report at 530 μm.^22^

**Figure 8:**
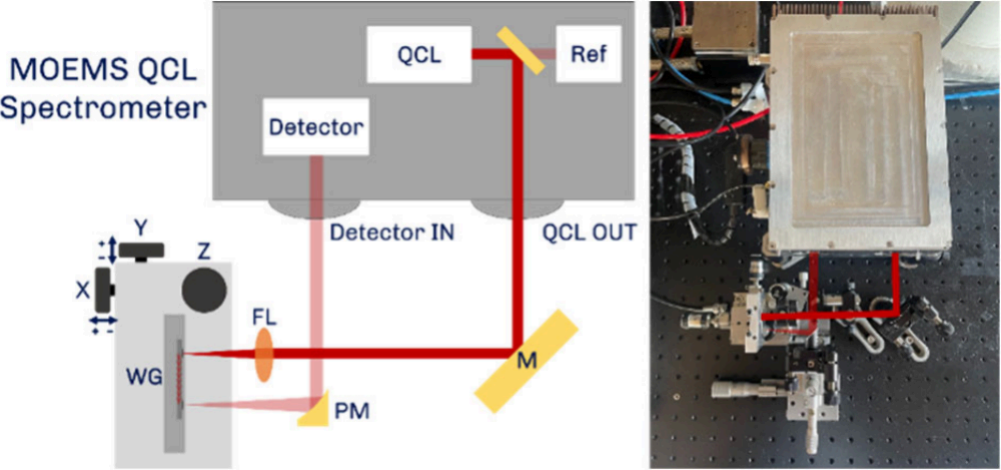
Schematic depiction of the setup to test the alignment tolerances of the waveguide platform (left); image of the setup (right).

For the testing of the waveguide platform, the chip was mounted on an *xyz*-stage equipped with manual adjusters (DS-4F, mks|Newport) with 1.33 μm graduations and a 300 μm fine travel range. The light source was a micro-opto-electro-mechanical system (MOEMS) external cavity quantum cascade laser (EC-QCL) developed in the European Union funded H2020 Project AQUARIUS. The laser was operated with a 570 kHz pulse repetition rate and a 100 ns pulse length and emitted between 6.36 and 7.45 μm. The used detector was a thermoelectrically cooled high speed MCT detector integrated (D* ≥ 4.0 × 10^9^ cm √Hz/W). The whole system is depicted in Figure 8.

Inside the spectrometer, the beam is split into the output beam and the reference beam using a 99/1 beamsplitter. The inherently linearly polarized QCL beam was adjusted to match the required propagating mode of the waveguide platform (designed for TE polarization). The collimated output beam is then directed on the focusing lens (FL), focusing the beam onto the integrated microlenses of the waveguide (WG) chip. After propagation, the out-coupled beam is collimated and directed toward the detector using an off-axis parabolic mirror (PM). The difference in path length between the reference beam and the beam used for sensing has to be noted, as the measurements were performed in air, and the presence of water vapor (having many absorption lines of their fundamental ro-vibrational transitions in the 6–7.5 μm wavelength range) led to additional noise which had to be considered in the evaluation of data by accounting for small fluctuations in water vapor concentration.

## Conclusion

We experimentally investigated the benefits of monolithically integrated microlenses for the backside coupling on our germanium-on-silicon trenched waveguide platform in a free space setup. For this, the chip was mounted on a an *xyz*-stage and an area of 100 μm × 100 μm was scanned in-plane centered at the point of maximum transmission. These measurements revealed a mean in-plane 1 dB alignment tolerance of the waveguide platform of ±16.4 μm, compared to a simulated grating bandwidth of ±3 μm. Out-of-plane measurements performed in a ±150 μm longitudinal 1D scan showed no significant losses. Further, the spectral features of the transmission spectra were investigated, showing that the spectral shifts caused by misalignments on the grating coupler can be reduced significantly. It was proven experimentally that employing a waveguide platform design with integrated microlenses leads to a very alignment tolerant system, going a step closer toward industrial application of GOS photonic sensing systems.

## References

[ref1] Soref R. A.; Emelett S. J.; Buchwald W. R. Silicon waveguided components for the long-wave infrared region*. J. Opt. A-Pure appl. Op. 2006, 8 (10), 840 10.1088/1464-4258/8/10/004.

[ref2] Amotchkina T.; Trubetskov M.; Hahner D.; Pervak V. Characterization of e-beam evaporated Ge, YbF3, ZnS, and LaF3 thin films for laser-oriented coatings. Appl. Opt. 2020, 59 (5), A40–A47. 10.1364/AO.59.000A40. 32225351

[ref3] Shkondin E.; Takayama O.; Panah M. E. A.; Liu P.; Larsen P. V.; Mar M. D.; Jensen F.; Lavrinenko A. V. Large-scale high aspect ratio Al-doped ZnO nanopillars arrays as anisotropic metamaterials. Opt. Mater. Express 2017, 7 (5), 1606–1627. 10.1364/OME.7.001606.

[ref4] Chang Y.-C.; Paeder V.; Hvozdara L.; Hartmann J.-M.; Herzig H. P. Low-loss germanium strip waveguides on silicon for the mid-infrared. Opt. Lett. 2012, 37 (14), 2883–2885. 10.1364/OL.37.002883. 22825166

[ref5] Roelkens G.; Dave U.; Gassenq A.; Hattasan N.; Hu C.; Kuyken B.; Leo F.; Malik A.; Muneeb M.; Ryckeboer E.; Uvin S.; Hens Z.; Baets R.; Shimura Y.; Gencarelli F.; Vincent B.; Loo R.; Van Campenhout J.; Cerutti L.; Rodriguez J.-B.; Tournié E.; Chen X.; Nedeljkovic M.; Mashanovich G.; Shen L.; Healy N.; Peacock A. C.; Liu X.; Osgood R.; Green W. Silicon-based heterogeneous photonic integrated circuits for the mid-infrared. Opt. Mater. Express 2013, 3 (9), 1523–1536. 10.1364/OME.3.001523.

[ref6] Shen L.; Healy N.; Mitchell C. J.; Penades J. S.; Nedeljkovic M.; Mashanovich G. Z.; Peacock A. C. Mid-infrared all-optical modulation in low-loss germanium-on-silicon waveguides. Opt. Lett. 2015, 40 (2), 268–271. 10.1364/OL.40.000268. 25679861

[ref7] Kang J.; Takenaka M.; Takagi S. Novel Ge waveguide platform on Ge-on-insulator wafer for mid-infrared photonic integrated circuits. Opt. Express 2016, 24 (11), 11855–11864. 10.1364/OE.24.011855. 27410108

[ref8] Younis U.; Luo X.; Dong B.; Huang L.; Vanga S. K.; Lim A. E.-J.; Lo P. G.-Q.; Lee C.; Bettiol A. A.; Ang K.-W. Towards low-loss waveguides in SOI and Ge-on-SOI for mid-IR sensing. J. Phys. Commun. 2018, 2 (4), 045029 10.1088/2399-6528/aaba24.

[ref9] Marris-Morini D.; Vakarin V.; Ramirez J. M.; Liu Q.; Ballabio A.; Frigerio J.; Montesinos M.; Alonso-Ramos C.; Le Roux X.; Serna S.; Benedikovic D.; Chrastina D.; Vivien L.; Isella G. Germanium-based integrated photonics from near- to mid-infrared applications. Nanophotonics 2018, 7 (11), 1781–1793. 10.1515/nanoph-2018-0113.

[ref10] Wietler T. F.; Bugiel E.; Hofmann K. R. Relaxed germanium films on silicon (110). Thin Solid Films 2008, 517 (1), 272–274. 10.1016/j.tsf.2008.08.018.

[ref11] Nedeljkovic M.; Penades J. S.; Mittal V.; Murugan G. S.; Khokhar A. Z.; Littlejohns C.; Carpenter L. G.; Gawith C. B. E.; Wilkinson J. S.; Mashanovich G. Z. Germanium-on-silicon waveguides operating at mid-infrared wavelengths up to 8.5 μm. Opt. Express 2017, 25 (22), 27431–27441. 10.1364/OE.25.027431. 29092216

[ref12] Malik A.; Muneeb M.; Radosavljevic S.; Nedeljkovic M.; Penades J. S.; Mashanovich G.; Shimura Y.; Lepage G.; Verheyen P.; Vanherle W.; Van Opstal T.; Loo R.; Van Campenhout J.; Roelkens G. Silicon-based Photonic Integrated Circuits for the Mid-infrared. Procedia Eng. 2016, 140, 144–151. 10.1016/j.proeng.2015.10.154.

[ref13] Gallacher K.; Millar R. W.; Griškevičiu̅te U.; Baldassarre L.; Sorel M.; Ortolani M.; Paul D. J. Low loss Ge-on-Si waveguides operating in the 8–14 μm atmospheric transmission window. Opt. Express 2018, 26 (20), 25667–25675. 10.1364/OE.26.025667. 30469665

[ref14] Montesinos-Ballester M.; Vakarin V.; Liu Q.; Le Roux X.; Frigerio J.; Ballabio A.; Barzaghi A.; Alonso-Ramos C.; Vivien L.; Isella G.; Marris-Morini D. Ge-rich graded SiGe waveguides and interferometers from 5 to 11 μm wavelength range. Opt. Express 2020, 28 (9), 12771–12779. 10.1364/OE.391464. 32403767

[ref15] Marchetti R.; Lacava C.; Carroll L.; Gradkowski K.; Minzioni P. Coupling strategies for silicon photonics integrated chips [Invited]. Photonics Res. 2019, 7 (2), 201–239. 10.1364/PRJ.7.000201.

[ref16] Taillaert D.; Bienstman P.; Baets R. Compact efficient broadband grating coupler for silicon-on-insulator waveguides. Opt. Lett. 2004, 29 (23), 2749–2751. 10.1364/OL.29.002749. 15605493

[ref17] Marchetti R.; Lacava C.; Khokhar A.; Chen X.; Cristiani I.; Richardson D. J.; Reed G. T.; Petropoulos P.; Minzioni P. High-efficiency grating-couplers: demonstration of a new design strategy. Sci. Rep. 2017, 7 (1), 16670 10.1038/s41598-017-16505-z. 29192215 PMC5709428

[ref18] Lee J.-M.; Kim K.-J.; Kim G. Enhancing alignment tolerance of silicon waveguide by using a wide grating coupler. Opt. Express 2008, 16 (17), 13024–13031. 10.1364/OE.16.013024. 18711541

[ref19] Mangal N.; Snyder B.; Van Campenhout J.; Van Steenberge G.; Missinne J. Expanded-Beam Backside Coupling Interface for Alignment-Tolerant Packaging of Silicon Photonics. IEEE J. Sel. Top. Quantum Electron. 2020, 26 (2), 1–7. 10.1109/JSTQE.2019.2934161.

[ref20] Missinne J.; Benéitez N. T.; Mangal N.; Zhang J.; Vasiliev A.; Campenhout J. V.; Snyder B.; Roelkens G.; Steenberge G. V. Alignment-tolerant interfacing of a photonic integrated circuit using back side etched silicon microlenses. Proc. SPIE 2019, 1092304 10.1117/12.2506159.

[ref21] Mangal N.; Snyder B.; Van Campenhout J.; Van Steenberge G.; Missinne J. Monolithic integration of microlenses on the backside of a silicon photonics chip for expanded beam coupling. Opt. Express 2021, 29 (5), 7601–7615. 10.1364/OE.412353. 33726258

[ref22] Benéitez N. T.; Baumgartner B.; Missinne J.; Radosavljevic S.; Wacht D.; Hugger S.; Leszcz P.; Lendl B.; Roelkens G. Mid-IR sensing platform for trace analysis in aqueous solutions based on a germanium-on-silicon waveguide chip with a mesoporous silica coating for analyte enrichment. Opt. Express 2020, 28 (18), 27013–27027. 10.1364/OE.399646. 32906963

